# 

**Published:** 1890-05

**Authors:** 


SUBSCRIBE FOR THE

### INTERNATIONAL DENTAL JOURNAL

FOR 1890.

Its fearless, independent course in defence of Professional interests should make it indispensable to every dentist.

ALL DEALERS ARE OUR AUTHORIZED AGENTS. TERMS, $2.50 PER ANNUM, IN ADVANCE.

